# Antidiarheal activity of catechol and ethyl 5, 8,11,14,17 – icosapentanoate-rich fraction of *Annona senegalensis* stem bark

**DOI:** 10.1016/j.jtcme.2021.07.007

**Published:** 2021-08-05

**Authors:** Maryam Usman Ahmed, Rotimi Olusanya Arise, Isaac John Umaru, Abdulrasheed Mohammed

**Affiliations:** a Department of Biochemistry, Adamawa State University, Mubi, Adamawa, Nigeria; b Department of Biochemistry, University of Ilorin, Ilorin, Nigeria; c Department of Biochemistry, Federal University, Wukari, Taraba State, Nigeria; d Department of Biochemistry, Abubakar Tafawa Balewa University, Bauchi, Nigeria

**Keywords:** Antidiarrheal, Na^+^-K^+^ ATPase, Cyclooxygenase II, Antioxidant, Lipid peroxidation, COX II, Cycloxygenase II, GPx, Glutathione peroxidase, SOD, Superoxide dismutase, DPPH, 2,2-diphenyl-1-picrylhydrazyl, ABTS, 2,2-azino-bis-3-ethylbenzothiazoline-6-sulfonic acid, MDA, Malondialdehyde, DS, Dichloromethane stem bark extract, HS, Hexane stem bark extract, AS, aqueous stem bark extract, DMSO, dimethylsufoxide, HFAS, Hexane fraction of aqueous stem bark extract, EFAS, ethylacetate fraction of aqueous stem bark extract, DFAS, diethylacetate fraction of aqueous stem bark extract, AFAS, aqueous fraction of aqueous stem bark extract

## Abstract

**Background and aim:**

Secretory diarrhea is the most common type of diarrhea. This study aimed at exploring the possible mechanism of antisecretory action of Annona senegalensis stem bark and to identify the bioactive compounds.

**Experimental procedure:**

The ability of three crude extract; aqueous, dichloromethane and hexane stem bark extracts to inhibit castor oil-induced stooling in albino rats were assessed. Bioactivity guided fractionation of the most active extract was done using solvent-solvent partitioning (with hexane, dichloromethane, ethylacetate) and column chromatography. In vitro antioxidant activity of the most active sub-fraction was done using standard methods. The most active sub-fraction (25 mg/kg b. wt.) was administered to castor oil-induced diarrheal rats. Diarrheal rats small intestinal malondialdehyde concentration, antioxidant enzyme, cyclooxygenase II and Na^+^- K^+^ ATPase activities were determined using standard procedures. GC-MS analysis was done to identify the chemical compounds in the sub-fraction.

**Result and conclusion:**

Aqueous extract significantly decreased the number of wet stools. Sub-fraction 1 of ethylacetate fraction of aqueous stem bark extract (EFAS1) showed the highest stool inhibition. The H_2_O_2_ scavenging activity of EFAS1 was significantly greater than ascorbic acid. The sub-fraction significantly increased (p < 0.05) the activity of catalase and Na^+^- K^+^ ATPase activities but significantly decreased the concentration of malondialdehyde and cyclooxygenase II activity. GC-MS analysis revealed that EFAS1 is rich in catechol, n-hexadecanoic acid and ethyl-5,8,11,14,17-icosapentanoate. The sub-fraction exerts its antisecretory activity by its antioxidative, inhibition of prostaglandin synthesis and stimulation of Na^+^- K^+^ ATPase properties due to the presence of catechol, n-hexedecanoic acid and ethyl-5,8,11,14,17-icosapentanoate.

## Highlights

• Antisecretory compounds identified are catechol, ethyl icosapentanoate and hexadecanoate.
• The antisecretory fraction obtained stimulates Na+-K^+^ ATPase activity.
• It has antioxidant activity and thus prevents lipid peroxidation.
• It inhibits prostaglandin synthesis by inhibiting COX II activity.

## Graphical abstract

**Figure:**
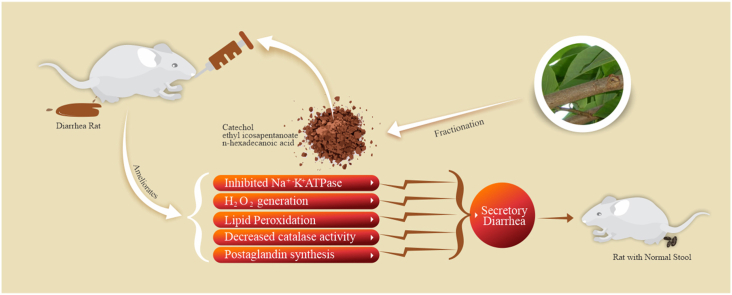

## 1 Introduction

Diarrhea is an abnormal bowel movement and it is characterized by an increase in the frequency and fluidity of stool.^1^ Secretory diarrhea is a type of diarrhea caused by net increase of chloride or bicarbonate and fluid into the lumen.^2^ The result of increase in electrolyte secretion is the decrease in the absorption of sodium and water.^2^ In secretory diarrhea, fluid secretion involves activation of cyclic nucleotides (cAMP and cGMP), multiple ion and solute transporters.^3^ . Diarrhea is usually managed with oral rehydration therapy (ORT) to reduce severe dehydration. Oral rehydration therapy does not actually stop diarrhea but will prevent dehydration until the causative agent is eradicated.^4^ It therefore follows that in cases of severe fluid losses, drugs that will target the causative agents must be administered. The side effects associated with some of the antidiarrheal therapies include drug toxicity, sedation, dizziness, dependency, respiratory depression, nausea and constipation.^5^

*Annona senegalensis* is commonly called African custard apple. The stem has been reported to have antidiarrheal property probably by its antisecretory potential.^6^ Several mechanisms are responsible for hypersecretion of water and electrolytes; prostaglandin synthesis, oxidative stress and hypersecretion of chloride ions. The particular mechanism through which *Annona senegalensis* stem bark exert its antisecretory property has not been documented. This paper seeks to fractionate the stem bark with the aim of identifying antisecretory compounds and to explore their possible mechanism of anti-secretory action.

## 2 Materials and methods

### 2.1 Collection and preparation of plants

*Annona senegalensis* stem barks were collected in July 2019 and authenticated at the Herbarium Unit, Plant Biology Department, University of Ilorin, Ilorin, Nigeria and was deposited at the University Herbarium. They were washed cleaned and air - dried under shade. The powdered samples were kept in airtight containers at room temperature until required for use.

#### 2.1.1 Extraction procedure

The powdered plant samples were soaked separately in three solvents; hexane, dichloromethane and water in the ratio 1: 10 for 24 h at 35 °C with vigorous shaking at 3 h intervals. Whatman No. 1 filter paper was used to filter the crude extract. Each of the filtrates was evaporated to dryness at 40 °C under reduced pressure and the dried substance was stored in airtight bottle until required.

### 2.2 Experimental animals

Adult albino rats of both sexes weighing between 130 and 150 g were obtained from the Animal Breeding Unit of the Department of Biochemistry, University of Ilorin, Ilorin, Nigeria. They were housed in well ventilated aluminum cages, and given standard laboratory diet and water *ad libitum.* The rats were handled according to the guidelines for the protection and handling of laboratory animals by the International Council for Laboratory Animal Science (ICLAS) and approved by the ethical committee of University of Ilorin and was given an approval number: UERC/ASN/2018/1216.

### 2.3 Evaluation of the antisecretory activity of *A. senegalensis* stem bark

#### 2.3.1 Induction of diarrhea

The rats were fasted for 18 h with free access to water. The rats were administered 1 mL castor oil orally using orogastric cannula to induce diarrhea.

#### 2.3.2 Inhibitory effect of solvent extracts of *A. senegalensis* stem bark on watery stool in castor oil-induced diarrhea

Twenty five albino rats were randomly divided into five groups for the antidiarrheal activity of crude extracts. Group I and II (diarrheal control) received 1.0 mL 20% tween and 1.0 mL 20% DMSO respectively, group III received 3.0 mg/kg b.wt. loperamide, groups IV - V received 100 mg/kg b. wt. dichloromethane stem bark extract (DS) and 100 mg/kg b. wt. hexane stem bark extract (HS) separately reconstituted in 20% DMSO while group VI received 100 mg/kg b. wt. aqueous stem bark extract (AS) reconstituted in 20% tween. After an hour of induction of diarrheal, the various extracts were administered. The animals were placed separately in metabolic cages over white clean Whatman filter paper, which was changed every hour. The total number of watery stool in each cage was recorded by inspecting the individual cages for the presence of characteristic diarrheal dropping. The severity of diarrhea was assessed for 4 h. The total number of diarrhea feces of the control group was considered 100%.

% inhibition = (Control - Test) × 100/Control

### 2.4 Bioactivity guided fractionation of the most active antisecretory crude extract of *A. senegalensis* stem bark

#### 2.4.1 Solvent-solvent partitioning of the most active antisecretory crude extract of *A. senegalensis* stem bark

Solvent-solvent partitioning was done using the method described by Van-Wagener et al.^7^ The fractions were collected and evaporated to dryness using a rotary evaporator at 40 °C. All fractions were collected and tested for stool inhibitory activity using a dose of 50 mg/kg b.wt. fraction. The fraction with the highest stool inhibition was selected as the most active fraction. The most active solvent fraction was subjected to partial purification.

#### 2.4.2 Partial purification of solvent fractions from aqueous *A. senegalensis* stem bark

The ratio of 1.0 g solvent fraction per 20 silica gel powder was used to determine the weight of the silica gel that was placed in a column. The column for the fractionation was 390 mm long with radius of 15 mm. The fraction was dissolved in the eluting solvent and transferred into the column. Elution was done using the solvent determined by thin layer chromatography; ethylacetate: butanol: water: acetic acid (50:40:5:5)] was used at a flow rate of 6 mL/min. Eluants were collected in 100 mL. The fractions were spotted on thin layer chromatography (TLC) plates. Sub-fractions with the same retention factor (Rf) in the TLC plate were pooled together. All sub-fractions were evaluated for stool inhibitory activity at a dose of 25 mg/kg b.wt sub-fraction. Sub-fractions with the same level of activity were pooled together as one main sub-fractions.

### 2.5 Evaluation of mechanism of action of the antisecretory sub-fraction

#### 2.5.1 *In vitro* antioxidant activity of the antisecretory sub-fraction

The antisecretory sub-fraction was screened for *in vitro* antioxidant activity by using the following antioxidant models; DPPH scavenging activity, ferric reducing antioxidant power, ABTS scavenging activity, hydroxyl radical, hydrogen peroxide and total antioxidant capacity.

#### 2.5.2 Preparation of intestinal homogenates

Ten albino rats were divided into two groups. Diarrhea was induced as described above. Group II received 25 mg/kg b.wt. antisecretory sub-fraction. The castor oil-induced diarrheal rats were sacrificed by ether anaesthesia after an hour of administration of antisecretory sub-fraction. The small intestine of each rat was carefully removed. The intestinal content was collected by milking into a test tube. The small intestine was homogenized in 0.25 M sucrose (1: 4).

#### 2.5.3 Biochemical analysis

The malondialdehyde (MDA) concentration, superoxide dismutase, catalase, glutathione peroxidase Na^+^- K^+^ ATPase and cyclooxygenase II (COX II) activities of the small intestine homogenates were determined according to standard methods.

### 2.6 Identification of compounds in the antisecretory sub-fraction

The identification of compounds in the antisecretory sub-fraction was performed on a GC-MS equipment.

### 2.7 Statistical analysis

The computation of the mean and statistical analysis was done using SPSS software version 24.0. Data are expressed as mean ± S.E.M of group of five determinations. Data were statistically analyzed using one-way analysis of variance and Duncan multiple range test. Results with p values < 0.05 was taken to imply statistical significance.

## 3 Results

### 3.1 Inhibitory effect of solvent extracts of *Annona senegalensis* stem bark on watery stool in castor oil-induced diarrhea

Table 1 shows the stool inhibition of solvent extracts of *A. senegalensis* stem bark in castor oil-induced diarrheal rats. Aqueous stem (AS) extract significantly decreased (p < 0.05) the number of watery feces when compared to its negative control (Tween). The extract (AS) had 100% inhibition of watery feaces and was comparable with the group treated with the standard drug (loperamide).

**Table 1:** Inhibitory effects of solvent extracts of *A. senegalensis* stem bark on number of watery stool in castor oil-induced diarrheal rats.

	Group I	Group II	Group III	Group IV	Group V	Group VI
	DMSO	Tween	Loperamide	100 mg/kg b.wt. DS	100 mg/kg b.wt. HS	100 mg/kg b.wt. AS
**No. of watery feces**	8 ± 0.88^e^	5 ± 0.33^d^	0 ± 0.00^a^	2 ± 0.05^c^	1 ± 0.07^b^	0 ± 0.00^a^
**% inhibition**	–	–	100.00	61.50	57.70	100.00

Values are mean of five replicates ± S.E.M. Values with different superscript down the column are significantly different (p < 0.05).

### 3.2 Bioactivity guided fractionation of aqueous *A. senegalensis* stem bark

Table 2 depicts the stool inhibition of solvent fractions obtained from aqueous stem bark extract of *A. senegalensis* in castor oil-induced diarrheal rats. The ethylacetate partitioned fraction (EFAS) significantly reduced (p < 0.05) the number of wet stools (1 ± 0.03) when compared with the negative control (4 ± 0.27). Table 3 shows the R_f_ values of each of the 9 sub-fractions obtained from column chromatography of EFAS. Sub-fractions 1–4 were pooled together based on the similarity in R_f_ values while sub-fractions 5–8 were also pooled together. Table 4 shows the stool inhibition of sub-fractions from ethylacetate fraction of aqueous extract of *Annona senegalensis* stem bark (EFAS) in castor oil – induced diarrheal rats. Sub-fractions 1 and 2 (EFAS1 and EFAS2) significantly reduced (p < 0.05) the number of watery stool to 0.00 ± 0.00 when compared to the negative control (3.33 ± 0.23).

**Table 2:** Inhibitory effect of solvent fractions obtained from aqueous stem bark extract of *A. senegalensis* on number of watery stool in castor oil-induced diarrheal rats.

	Control	Loperamide	50 mg/kg b.wt HFAS	50 mg/kg b.wt. EFAS	50 mg/kg b.wt. DFAS	50 mg/kg b.wt. AFAS
**No. of watery feces**	4 ± 0.27^c^	0 ± 0.00^a^	4 ± 0.12^c^	1 ± 0.03^a^	3 ± 0.23^b^	2 ± 0.18^b^
**% inhibition**	–	100.00	0.00	83.25	33.25	50.00

Values are mean of five replicates ± S.E.M. Values with different superscript down the column are significantly different (p < 0.05).

HFAS- Hexane fraction of aqueous stem bark extract, EFAS – ethylacetate fraction of aqueous stem bark extract, DFAS- diethylacetate fraction of aqueous stem bark extract, AFAS- aqueous fraction of aqueous stem bark extract.

**Table 3:** Retention factor (R_f_) of sub-fractions from ethylacetate fraction of aqueous extract of *A. senegalensis* stem bark (EFAS).

Sub-fraction	R_f_
EFAS1	0.51
EFAS2	0.51
EFAS3	0.52
EFAS4	0.51
EFAS5	–
EFAS6	–
EFAS7	–
EFAS8	–
EFAS9	0.19

**Table 4:** Inhibitory effect of sub-fractions from EFAS on number of watery stool in castor oil - induced diarrheal rats.

	Control	25 mg/kg b.wt EFAS1	25 mg/kg b.wt EFAS2	25 mg/kg b.wt EFAS3
**No. of watery feces**	3.33 ± 0.23^c^	0.00 ± 0.00^a^	0.00 ± 0.00^a^	2.67 ± 0.21^b^

Values are mean of five replicates ± S.E.M. Values with different superscript down the column are significantly different (p < 0.05).

### 3.3 Evaluation of the mechanism of action of the antisecretory sub-fraction (EFAS1)

**Fig. 1:**
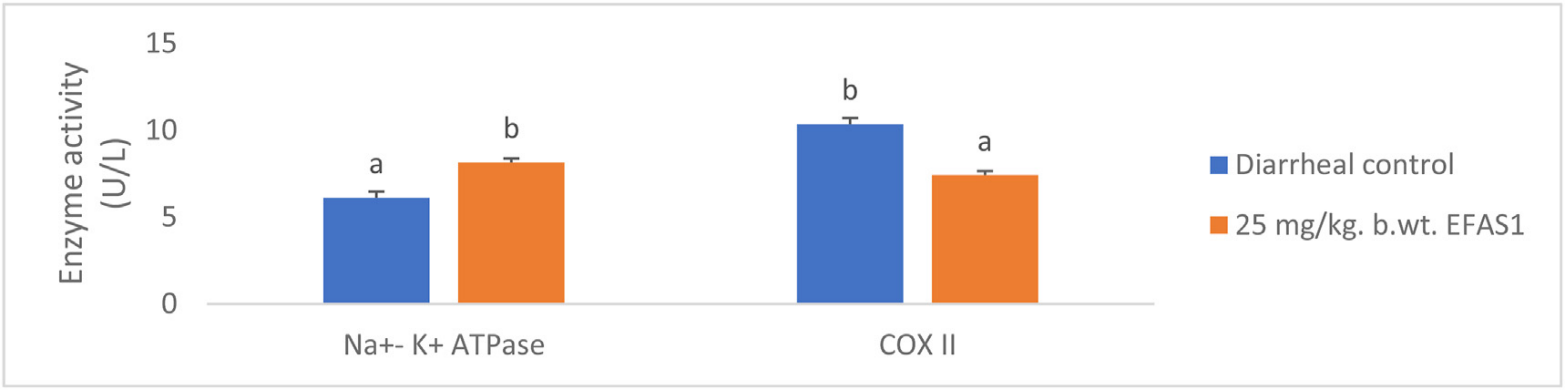
Effect of administration of EFAS1 on small intestinal Na^+^-K^+^ ATPase and cyclooxygenase II (COX II) activities of castor oil-induced diarrheal rats values are mean of five replicates ± S.E.M. Bars with different superscript in each category are significantly different (p < 0.05).

The result of antioxidant activity of EFAS1 is presented in Supplementary Table 5. The H_2_O_2_ scavenging activity of EFAS1 was significantly greater (p < 0.05) than that of ascorbic acid. Supplementary Table 6 shows the small intestinal antioxidant enzymes activity and concentration of malondialdehyde of EFAS1 in castor oil-induced diarrheal rats. The sub-fraction significantly increased (p < 0.05) the activity of catalase when compared with the control. There was no significant difference (p > 0.05) in the glutathione peroxidase (GPx) concentration of rats administered EFAS1 when compared with the diarrheal control. The sub-fraction significantly decreased (p < 0.05) the activity of superoxide dismutase (SOD) and reduced glutathione (GSH) when compared with the control. There was significant decrease (p < 0.05) in the small intestinal concentration of MDA of rats administered EFAS1. The sub-fraction EFAS1 significantly increased (p < 0.05) the activity of Na^+^- K^+^ ATPase when compared to the control and significantly decreased (p < 0.05) the activity of COX II as shown in Fig. 1.

### 3.4 Identification of compounds in EFAS1

Supplementary Fig. 2 shows the GC-MS chromatogram of EFAS1*.* Supplementary Table 7 shows the identified compounds in sub-fraction 1 of ethylacetate fraction of aqueous stem bark extracts (EFAS1) of *A. senegalensis.* GC-MS analysis of EFAS1 showed 30 peaks with different retention time (RT) values. Twenty one (21) compounds were identified. Catechol was identified with RT values between 6.712 and 6.941 as shown in Supplementary Table 7. Other compounds identified are hexadecanoic acid, kaur-16-ene, ethyl 5,8,11,14,17, icosapentanoate, 2,4,5, pyrimidinetriamine and androstan-3-17-dione 9,11, epoxy with peak area as 1.34%, 1.10%, 19.15%, 43.89%, 2.16% and 0.41% respectively.

## 4 Discussion

Generally, reduction in wet stool suggests the ability of the extract to inhibit hyper-secretion of electrolytes and fluid.^8^ The significant reduction in the number of watery stools by EFAS1 indicates that this sub-fraction contains substances that have antisecretory activity. The efficient H_2_O_2_ scavenging activity of EFAS1 shows that EFAS1 possess high antioxidant activity. . Reactive oxygen species has been implicated in diarrhea due to its ability to induce oxidative damage of lipids. The ability of this sub-fraction to scavenge for reactive oxygen species imply that it can prevent the harmful effects of reactive oxygen species produced *in vivo*. Oxidative stress has been reported to be involved in intestinal hypersecretion.^9^ Thus, the antioxidant activity of EFAS1 contributes to the anti-secretory potential of this sub-fraction and eventually improvement in diarrhea.

Castor oil-induced diarrhea cause a depletion of catalase and glutathione peroxidase (GPx) activities which results in oxidative stress.^10^ Increased catalase activity observed in this study therefore, provides a first line defense system against intestinal hypersecretion caused by oxidative stress. Superoxide radicals is one of the major ROS generated in the intestine.^9^ The significant reduction in superoxide dismutase activity in rats administered EFAS1 may be attributed to decrease generation of superoxide radicals since elevated level of superoxide radical increases the cellular concentration of superoxide dismutase. Increased superoxide dismutase activity has been reported to correlate with an increase in castor oil induced fluid accumulation.^11^ Therefore, the reduction in superoxide dismutase activity observed, will lead to a decrease in castor oil-induced intraluminal fluid accumulation; contributing to the antisecretory potential of this sub-fraction.

Castor oil increases the formation of malondialdehyde in the gastrointestinal mucosa due to oxidative stress indicating an increase in lipid peroxidation.^12^ Decreased concentration of MDA observed in this study, indicates the ability of EFAS1 to inhibit lipid peroxidation. This effect can be attributed to the ability of EFAS1 to scavenge effectively for H_2_O_2_. Hydrogen peroxide generation accompanies intestinal hypersecretion in the mucosal intestine.^12^ Effective H_2_O_2_ scavenging activity will protect against oxidative damage of lipids. Inhibition of lipid peroxidation by EFAS1 prevents intestinal barrier dysfunction and prevents the excessive production of arachidonic acid which will in turn prevent prostaglandin synthesis (a secretory agent).

The sodium pump plays an important role in the regulation of electrolyte and water fluxes in the intestinal mucosa.^13^ Na ^+^ - K^+^ ATPase activity decreases in all types of diarrhea.^14^ The significant increase in Na ^+^ - K^+^ ATPase activity by EFAS1 indicates that this sub-fraction may contain substances that are capable of restoring the electrochemical gradient by stimulating Na^+^ - K^+^ ATPase activity. The significant decrease in the activity of cyclooxygenase II in rats administered EFAS1 indicates that they are inhibitors of prostaglandin synthesis. Prostaglandins, formed through cyclooxygenase pathway, stimulates secretion of chloride ion resulting in diarrhea.^15^

Catechol, one of the major constituents of EFAS1 fraction, is a component of catecholamine. Catechol is a flavonoid. Flavonoids inhibit intestinal motility and hydroelectrolytic secretions.^16^ Catecholamine, an organic compound that has a catechol ring and amine, stimulates electrogenic NaCl absorption and decrease electrogenic Cl^−^ secretion by interaction with α-adrenoreceptors on enterocyte.^17^ It acts at the α-adrenergic receptor coupled with the G-proteins to antagonize cAMP production. Donowitz et al.^18^ reported that the catechol moiety is important for maximal agonist activity at the α-adrenoreceptors. Fluid secretion in many secretory diarrheas is caused by activation of chloride channels by cyclic nucleotides such as cAMP.^3^ The most obvious target for anti-secretory therapy are the second messengers such as cAMP.^19^ Thus, EFAS1 ameliorate secretory diarrhea through the presence of catechol which antagonizes cAMP production.

EFAS1 also contains 1.34% n-hexadecanoic acid. n-hexadecanoic acid is a competitive inhibitor of phospholipase A_2_.^20^ n-hexadecanoic acid is a competitive inhibitor of phospholipase A_2_. Inhibition of phospholipase A_2_ (PLA_2_) depletes the downstream arachidonic acid metabolites and thus, inhibit cyclooxygenase (COX) II.^21^ Therefore, the n-hexadecanoic acid in EFAS1 is responsible for the inhibition of COX II activity. The major constituent of EFAS 1, ethyl 5,8,11,14,17, icosapentanoate is an unsaturated fatty acid ester. . Silva et al.^22^ reported that unsaturated fatty acid is responsible for the activation of Na ^+^ - K^+^ ATPase activity. Thus, ethyl icosapentanoate is responsible for the activation of Na^+^- K^+^ ATPase activity.

## 5 Conclusion

Aqueous stem bark extract contains anti-secretory compounds present in the ethylacetate fraction. The sub-fraction is rich in catechol and ethyl 5,8,11,14,17, icosapentanoate. Another anti-secretory compound present in the fraction is n-hexadecanoic acid. The mechanism of action of the antisecretory fraction is antioxidative, prostaglandin synthesis inhibition and stimulation of Na^+^- K^+^ ATPase activity. These anti-secretory compounds act synergistically to prevent wet stool characterized by diarrhea.

## Declaration of competing interest

All authors declare that there is no conflict of interest. The research was not supported by any form of funding nor grant.

## Footnotes

Appendix A Supplementary data to this article can be found online at https://doi.org/10.1016/j.jtcme.2021.07.007.
