# Supplementary material for: Antidiarheal activity of catechol and ethyl 5, 8,11,14,17 – icosapentanoate-rich fraction of Annona senegalensis stem bark
Source: J Tradit Complement Med. 2021 Aug 5;12(2):190–4. doi: 10.1016/j.jtcme.2021.07.007 (PMC9072799; doi:10.1016/j.jtcme.2021.07.007)
Supplement: Multimedia component 1 [file mmc1.docx]

**SUPPLEMENTARY MATERIAL**

Table 5: Antioxidant activity of the sub-fraction EFAS1 obtained from *A. senegalensis* stem barks

|  | **DPPH (IC_50_)**  **(mmol/L)** | **FRAP (nmol/L)** | **ABTS (IC_50_)**  **(mmol/L)** | **H_2_O_2_ (mol/L)** | **TAC (tannic acid equiv.)** | **OH^-^ (mol/L)** |
| --- | --- | --- | --- | --- | --- | --- |
| Ascorbic | 12.09 ± 0.18^a^ | 127.67 ± 0.98^a^ | 33.92 ± 0.80^a^ | 6.32 ± 0.31^a^ | 150.67 ± 1.23^b^ | 70.20 ± 0.09^b^ |
| EFAS1 | 24.42 ± 1.95^b^ | 127.83 ± 1.06^a^ | 63.89 ± 1.68^b^ | 7.17 ± 0.06^b^ | 121.99 ± 3.93^a^ | 64.89 ± 0.37^a^ |

Values are mean of five replicates ± S.E.M. Values with different superscript down the column are significantly different (p < 0.05).

Table 6: Effect of administration of EFAS1 on small intestinal antioxidant enzyme activities and malondialdehyde (MDA) concentration of castor oil- induced diarrheal rats

| **Groups** | **Catalase (U/L)** | **GPx (U/L)** | **SOD (U/L)** | **MDA (mg/dL)** |
| --- | --- | --- | --- | --- |
| Diarrhoeal conttrol | 2.28 ± 0.19^a^ | 5.59 ± 0.68^a^ | 8.01 ± 0.31^b^ | 12.47 ± 0.12^b^ |
| 25 mg/kg. b.wt. EFAS1 | 7.60 ± 0.87^b^ | 5.61 ± 0.21^a^ | 6.38 ± 0.17^a^ | 9.20 ± 0.17^a^ |

Values are mean of five replicates ± S.E.M. Values with different superscript down the column are significantly different (p < 0.05)

Figure 2: GC-MS chromatogram of sub-fraction 1 of ethylacetate fraction from aqueous ethylacetate stem bark extract (EFAS1) of *A. senegalensis*

Table 7: Chemical compounds present in sub-fraction 1 of ethylacetate fraction from aqueous stem bark extract (EFAS1) of *Annona senegalensis*

| **Compound name** | **RT(min)** | **% peak area** | **Class of compound** |
| --- | --- | --- | --- |
| Catechol | 6.712 | 7.78 | Phenol |
| Catechol | 6.746 | 2.83 | Phenol |
| Catechol | 6.775 | 2.78 | Phenol |
| Catechol | 6.821 | 1.35 | Phenol |
| Catechol | 6.838 | 1.94 | Phenol |
| Catechol | 6.889 | 1.53 | Phenol |
| Catechol | 6.941 | 0.49 | Phenol |
| Phenol, 3,4,5, trimethyl | 8.064 | 0.05 | Flavonoid |
| 3-(1-hydroxy-1-methyl-ethyl) 5 - phenyl isoxazolin -3-ol | 8.796 | 1.07 | Heterocyclic |
| 6,7, dimethyl triazolo (4,3, -b, 1,2,4) triazine | 9.830 | 0.94 | Heterocyclic nitrogen compound |
| n-hexadecanoic acid | 10.929 | 1.34 | Fatty acid |
| Diazoprogesterone | 11.026 | 0.28 | Steroid |
| Kaur-16-ene | 11.187 | 0.33 | Diterpene alkaloid |
| Trachylobane | 11.547 | 0.35 | Tetracyclic diterpenes |
| Hexane, 1-chloro-5-methyl | 11.656 | 1.10 | Tetracyclic diterpenes |
| Kaur-16-ene | 11.856 | 6.33 | Diterpene alkaloids |
| Kaur-16-ene | 11.873 | 12.82 | Diterpene alkaloids |
| 3,6, Nonadien-1-ol | 12.480 | 0.47 |  |
| 1,8, Nonadiene, 2 methyl 5,7, dimethylene | 12.686 | 4.13 | Alkene |
| Ethyl 5,8,11,14,17, icosapentaenoate | 12.835 | 43.89 | Fatty acid |
| 2,4,5 pyrimidinetriamine | 13.899 | 2.16 |  |
| Androstan-3,17 dione 9,11, epoxy- | 13.950 | 0.41 | Steroid epoxy |
